# Medical Society of London

**Published:** 1854-01

**Authors:** 


Medical Society of London.—Dr. Crisp exhibited a dog, the spleen of which was removed two years and a half since, by Dr. Leared, of Finsbury-circus. The animal was in good condition, and did not appear in any way to have suffered from the loss of the organ. The blood, which was exhibited under the microscope, presented no abnormal appearance.

